# The Effects of Obesity-Related Health Messages on Explicit and Implicit Weight Bias

**DOI:** 10.3389/fpsyg.2016.02064

**Published:** 2017-01-11

**Authors:** Almut Rudolph, Anja Hilbert

**Affiliations:** Integrated Research and Treatment Center AdiposityDiseases, Department of Medical Psychology and Medical Sociology and Department of Psychosomatic Medicine and Psychotherapy, University of Leipzig Medical CenterLeipzig, Germany

**Keywords:** weight stigma, weight bias, explicit weight bias, implicit weight bias, health messages

## Abstract

The pervasiveness of explicit and implicit weight bias (WB) defined as negative stereotypes and prejudice regarding one’s weight has been observed among individuals of all weight categories. As a source of WB, health messages have been discussed due to reinforcing stigmatizing notions. The present study sought to investigate whether health messages (i.e., eat healthy, become physically active) have the potential to increase explicit and implicit WB. Participants (*N* = 144) from the community were randomized to either an experimental group (EG) or a control group (CG). While the EG was presented with health messages, the CG was presented with neutral information. Before and after manipulation, participants completed measures of explicit and implicit WB. Paired samples *t*-test revealed no differences in explicit WB after manipulation, however, a small effect decrease of implicit WB in the EG but not in the CG was found. This study provided evidence that health messages might have differential impact to change WB. According to dual-model approaches, explicit and implicit WB tap into two different information processing systems, and thus were differentially affected by health messages. Brief exposure to health messages might have the potential to contribute to health behavior and to mitigate implicit WB.

## Introduction

Overweight and obese individuals experience pervasive WB, defined as negative stereotypes and prejudice regarding their weight, in multiple domains of life, for example, in employment, in educational and health care settings, in the media as well as in interpersonal relationships (Puhl and Heuer, 2009; Puhl and King, 2013). Consequently, experiencing WB has been strongly associated with psychosocial maladjustment and poor weight loss outcome (Carels et al., 2010). WB has been observed using self-report questionnaires (i.e., explicit WB; Allison et al., 1991), and computerized measures (i.e., implicit WB; Phelan et al., 2014).

Dual-model approaches (e.g., Strack and Deutsch, 2004) provide an elaborated theoretical framework comprising two structurally distinct systems of cognitive processing that encompasses explicit and implicit evaluations. Thus, explicit WB is understood as deliberative, accessible and mentally represented information in a reflective system that can be assessed with self-report questionnaires, i.e., resulting in higher levels of agreement with items such as “Obese individuals are lazy, incompetent, and weak.” Implicit WB, however, is the automatic and not necessarily conscious evaluation in an impulsive system that can be assessed with implicit measures such as the Implicit Association Test (IAT; Greenwald et al., 1998). In a WB-IAT, respondents are required to co-classify stimuli in target categories (i.e., *Obese* vs. *Thin*) or attribute categories (i.e., *Positive* vs. *Negative*). They typically find responding easier – and hence go faster – when the categories are configured one way (e.g., *Obese* with *Negative*) rather than another (e.g., *Obese* with *Positive*). Furthermore, dual-process approaches specify how behavioral schemata such as discriminating behaviors might be activated by input from the two systems. In the reflective system, a behavioral option is weighted and integrated by the value and the probability of its potential consequences. In the impulsive system, behavioral schemata are triggered through spreading activation in the associative store without the need of deliberation and introspection. Whereas critics find fault with the arbitrary metric of the IAT (e.g., Blanton and Jaccard, 2006), previous research revealed typically weak correlations between explicit and implicit measures corroborating the theoretical dual-model framework (Hofmann et al., 2005; Brauhardt et al., 2014; Rudolph and Hilbert, 2014). Furthermore, several studies have documented the reliability of the IAT (e.g., Rudolph et al., 2008), and the predictive validity of implicit measures over and above self-report measures (Dovidio et al., 2002; Rudolph et al., 2010; Brauhardt et al., 2014).

Some authors argue that public health campaigns targeting obesity-related themes might be a source of WB due to reinforcing stigmatizing notions (Puhl et al., 2012, 2013). In fact, health messages have been publicly criticized for their stigmatizing content, and first empirical evidence exists that obesity-related public health campaigns have been perceived as stigmatizing (Puhl et al., 2013). The influence of public health messages on explicit and implicit WB, however, has not been investigated yet. Therefore, in a randomized-controlled study, we aimed to investigate whether information on health behavior as promoted in ubiquitous public health campaigns (i.e., eat healthy, become physically active) influence explicit and implicit WB. In our exploratory study, we expect that reading health messages – albeit neither explicitly focusing on controllable or uncontrollable causes of obesity but targeting obesity-related themes – will be associated with higher levels of explicit and implicit WB (Teachman et al., 2003; Crandall and Reser, 2005; O’Brien et al., 2010).

## Materials and Methods

### Participants

Participants (ages ≥ 18 years) were recruited through public advertisements (e.g., bulletin boards). They were provided with a link to the web-based survey and were offered to take part in a lottery (€10) upon completion as reimbursement. The study was introduced as a psychological examination investigating attitudes toward health. A total of 195 individuals consented to participate, however, 22% (*N* = 43) of them were excluded because of incomplete data. Completers and non-completers did not differ in age or gender (all *p* > 0.05). Additionally, 5% (*N* = 9) of the participants were excluded because their responses indicated careless participation, particularly, five participants were going too fast (i.e., more than 10% of test trials < 300 ms) and/or too erroneous (i.e., >30% false responses in the test trials) in the implicit measure (Nosek et al., 2007), and four participants did not complete the experimental procedure within a reasonable amount of time (i.e., pausing > 60 min during the survey).

### Measures

The *Beliefs About Obese Persons* (BAOP; Allison et al., 1991) *Scale* was used to assess explicit WB through the extent of agreement or disagreement (+3 to -3) regarding eight specific statements on causes of obesity. Respective items were inverted and 24 were added to the sum scores (range: 0–48). Lower BAOP scores indicated that participants preferred overeating and personal control as causes of obesity whereas lower scores indicated that participants preferred genetics and environment as causes of obesity.

The *Attitudes About Obese Persons* (ATOP; Allison et al., 1991) *Scale* was used to assess explicit WB through the extent of agreement or disagreement (+3 to -3) regarding 20 specific statements on positive and negative attitudes toward obese individuals. Respective items were inverted and 60 were added to the sum scores (range: 0–60). Lower ATOP scores indicated more negative attitudes toward obese individuals (i.e., strong WB).

The *Affective Priming Task* (APT; Fazio et al., 1995), along with the IAT (Greenwald et al., 1998), is a prominent as well as reliable and valid measure of implicit evaluations (Roefs et al., 2011), that assesses implicit WB as the degree to which a thin vs. fat prime stimuli facilitates judgments of positive vs. negative target stimuli. Prime stimuli were eight full body pictures of a normal weight and an obese woman and man presented from different angles using a virtual simulator. Target stimuli were 10 positive and negative valenced stimuli chosen during pre-test from the International Affective Picture System (Lang et al., 2008). In all trials, fixation cruces were presented (500 ms) followed by masked prime stimuli (67 ms). With a constant stimulus onset asynchrony (100 ms), target stimuli were presented (33 ms) with intertrial intervals of 1000 ms. Participants were asked to classify target stimuli according to their valence using one of two response keys. A practice block of 21 trials preceded the test blocks with a total of 160 trials (i.e., each prime stimuli paired twice with each target stimuli). Natural logarithmized response latencies were used to calculate implicit WB indices. Difference scores were calculated from the average logarithmized response latencies of the correct trials in incongruent prime – target pairings (i.e., thin – negative, fat – positive) and congruent prime – target pairings (i.e., thin – positive, fat – negative). Higher scores indicated a stronger preference for fat – negative over fat – positive (i.e., strong WB).

### Procedures

The study was approved by the ethical review board of the University of Leipzig (Ethik-Kommission an der Medizinischen Fakultät der Universität Leipzig). Given the web-based format, participants gave their informed consent via mouse click after reading the consent form that was initially presented. After giving consent, participants were randomly assigned to either an EG or a CG. Experimental manipulation comprised two different texts that were presented with equal structure (four paragraphs presented on two pages) and total length (360 words). The EG was presented with information on making behavioral changes to enhance health, i.e., increasing healthy food (“Healthy eating means consuming the right quantities of foods from all of the main food groups: whole grains, fruit and vegetables, protein, diary, and fat/sugar. A healthy and balanced nutritional lifestyle promotes good health, and includes […]. Following a healthy lifestyle can help you to prevent overweight and obesity: Eat healthy!”), and increasing physical activity (“Regular physical activity is important to maintain your health, increase your well-being, and reduce your risk of health problems. For a healthy lifestyle, a minimum of 30 min of moderate-intensity physical activity, e.g., walking, is recommended per day. […] Following a healthy lifestyle can help you to prevent overweight and obesity: Exercise regularly!”). The CG was presented with information about landscapes (e.g., geology, climate). This part of the study was introduced as a comprehension task that was designed to examine the comprehensibility of brochures. In order to ensure and strengthen information processing, participants wrote a summary about the presented content. Before and after the manipulation, participants completed APT, ATOP, and BAOP. Finally, participants reported their body weight and height. Self-reports and experimental manipulation were presented using Unipark Software, the APT was administered using Millisecond Software^TM^ with redirection between both programs using unique alphanumeric codes.

### Data Analysis

Descriptive statistics, X^2^ tests and independent samples *t*-test were conducted to characterize the sample. Pearson correlations determined relations between measures of WB. Independent samples *t*-test and paired samples *t*-test were run to assess between-group differences and within-group differences in WB scores before and after manipulation, respectively. Effect sizes *d* were calculated (small ≥ 0.20, medium ≥ 0.50, and large ≥ 0.80) (Cohen, 1988).

## Results

The final sample comprised 144 participants (110 women, 76.4%) with an average age of 28.72 ± 9.05 years. The BMI was 24.91 ± 7.14 kg/m^2^ with 66.7% underweight and normal weight participants (*N* = 96), and 33.3% overweight and obese participants (*N* = 48). EG and CG did not differ in gender, age, or BMI (all *p* > 0.05). Moreover, explicit and implicit measures of WB were not correlated (*r*_ATOP_APT_ = 0.03, *r*_BAOP_APT_ = -0.03; both *p* > 0.05).

### Between-Subjects-Analyses

Independent samples *t*-test revealed no differences for explicit WB, however, a small effect between-groups difference in APT scores were found [*t*(142) = -1.69, *p* = 0.09, *d* = 0.28]. Thus, EG and CG differed in implicit WB after manipulation with lower APT scores in the EG group than in the CG group (see **Table 1**).

**Table 1:** Mean and standard deviation of explicit and implicit WB indices before and after manipulation.

	Experimental group (*N* = 71)		Control group (*N* = 73)		Independent samples *t*-test				Paired samples *t*-test			
	Before manipulation	After manipulation	Before manipulation	After manipulation	Before manipulation		After manipulation		Experimental group		Control group	
	*M (SD)*	*M (SD)*	*M (SD)*	*M (SD)*	*t*(142)	*d*	*t*(142)	*d*	*t*(70)	*d*	*t*(72)	*d*
ATOP	70.31 (15.54)	69.90 (15.51)	71.00 (11.75)	70.64 (12.95)	-0.28	0.05	-0.31	0.05	0.45	0.03	0.37	0.03
BAOP	18.13 (8.30)	17.15 (7.60)	18.00 (7.53)	17.79 (7.95)	0.10	0.02	-0.49	0.08	1.94	0.12	0.42	0.03
APT	0.03 (0.09)	0.01 (0.06)	0.03 (0.05)	0.03 (0.06)	-0.47	0.08	-1.69	0.28	1.76	0.23	-0.46	0.08

*N = 144. APT, Affective Priming Task; ATOP, Attitudes About Obese Persons, Independent samples t-test to determine between-group differences in explicit and implicit WB indices before and after manipulation, paired samples t-test to assess within-group differences in explicit and implicit WB indices before and after manipulation, *d* = Cohen’s *d* (small effect ≥ 0.20, medium effect ≥ 0.50, large effect ≥ 0.80)*

### Within-Subjects-Analyses

No differences in explicit WB were found, however, paired samples *t*-test (see **Table 1**) revealed a small effect decrease of APT scores in the EG but not in the CG [*t*(70) = 1.76, *p* = 0.08, *d* = 0.23]. Therefore, as a result of reading health messages, participants were less likely to prefer fat – negative over fat – positive (i.e., decreased implicit WB).

## Discussion

This experimental study examined the influence of stigmatizing content of health messages on explicit and implicit WB. Contrary to our exploratory assumptions, exposure to obesity-related health messages emphasizing lifestyle changes did not strengthen people’s beliefs that overeating and a lack of personal control causes obesity (i.e., BAOP scores) nor their negative attitudes toward obese persons (i.e., ATOP scores), however, health messages *decreased* implicit WB.

The current study might have revealed a rather puzzling pattern of results, however, results should be interpreted in terms of two structurally distinct systems of information processing (Strack and Deutsch, 2004). First, and corroborating previous literature on the distinctiveness of the reflective and impulsive system (Strack and Deutsch, 2004; Brauhardt et al., 2014), explicit and implicit WB were found to be unrelated. Second, distinct routes for changing explicit and implicit evaluations have been proposed (Brinol et al., 2006). Thus, although the present study was not specifically designed to address mechanisms underlying these routes, we offer some insights. Explicit and implicit attitudes might be differentially responsive to deliberative and associative information, thus, whereas changes in explicit attitudes require some degree of motivational and attentional resources, implicit attitudes primarily change when attitude objects are paired with relevant positive or negative stimuli (Baccus et al., 2004; Strack and Deutsch, 2004; Rydell and McConnell, 2006). In the reflective system, brief exposure to health messages might have corroborate individuals’ believes that obesity is under the control of the obese person but might have failed to activate motivational and attentional resources for deliberative attitude changes (Strack and Deutsch, 2004). Previously, explicit attitude changes either occurred when audio-visual material was presented for a longer period of time (Domoff et al., 2012), or when messages were framed as related to the attitude object (Brinol et al., 2006). In the current study, however, the experimental manipulation comprised brief paragraphs of written words, and was introduced as a task designed to measure comprehensibility of new information rather than a task designed to measure persuasiveness of information. In the impulsive system, however, health messages might have automatically activated associated information (Strack and Deutsch, 2004) such as individuals’ efforts to maintain healthy lifestyle behaviors in participants’ everyday life, and these subsequently affected reactions in implicit measures. Previously, self-involvement was found to be an important factor in reducing implicit bias (Marini et al., 2012).

The major strength of the study is the randomized design, however, further research avenues may follow from the limitations of our study. First, replication of the results with a larger sample is warranted. Although, a more heterogeneous community sample than previous studies examining changes in WB (O’Brien et al., 2010; Domoff et al., 2012) was provided, the sample of the current study was not representative. A higher number of overweight and obese participants would further allow investigating WB changes in different BMI categories as perceptions of health messages were found to vary with participants’ body weight (Puhl et al., 2013). Second, limitations include the reliance on self-reported weight and height of the participants, which might have resulted in an underestimation of BMI (Gorber et al., 2007). Third, changes in WB were measured with less than 1 h between before and after manipulation assessment. Thus, replication of effects and their temporal stability are warranted to indicate whether changes in implicit WB are meaningful. Finally, future experimental research should examine whether health messages interfere with interventions to reduce WB. Previously, limited evidence for effective approaches to reduce WB has been reported (Danielsdottir et al., 2010; O’Brien et al., 2010), and public health campaigns have been criticized for their stigmatizing content (Syme, 2007; Puhl and Heuer, 2009).

## Conclusion

The authors suspect that understanding the differential relation between health messages and WB will shed light on the psychological mechanisms of the pervasiveness of explicit and implicit WB.

## Author Contributions

AR and AH conceptualized the research question and designed the study. AR analyzed the data. AR drafted the manuscript with critical comments from AH. All authors read and approved the final manuscript.

## Conflict of Interest Statement

The authors declare that the research was conducted in the absence of any commercial or financial relationships that could be construed as a potential conflict of interest.

## Abbreviations

CG: control group
EG: experimental group
WB: weight bias
